# Transmissibility of Clade IIb Monkeypox Virus in Young Rabbits

**DOI:** 10.3390/microorganisms13092182

**Published:** 2025-09-18

**Authors:** Zhaoliang Chen, Lei Zhang, Linzhi Li, Mingjie Shao, Mingda Zhang, Zongzheng Zhao, Chao Shang, Zirui Liu, Juxiang Liu, Zhendong Guo

**Affiliations:** 1 College of Veterinary Medicine, Hebei Agricultural University, 2596 Lucky South Street, Baoding 071000, China; czllight@163.com (Z.C.); 19832229138@163.com (M.Z.); 2 State Key Laboratory of Pathogen and Biosecurity, Changchun Veterinary Research Institute, Chinese Academy of Agricultural Sciences, 573 Tulip Street, Changchun 130122, China; leizhang9811@163.com (L.Z.); linzhili53@163.com (L.L.); mingshao165@163.com (M.S.); zzzfusheng@163.com (Z.Z.); shangchao1290@126.com (C.S.); killmarsking@163.com (Z.L.); 3 School of Life Science and Engineering, Southwest University of Science and Technology, 59 Middle Section, Qinglong Avenue, Mianyang 621010, China; 4 College of Life Sciences, Jilin Agricultural University, 2888 Xincheng Street, Changchun 130118, China

**Keywords:** monkeypox virus, aerosol shedding, contact transmission, airborne transmission, young rabbit

## Abstract

The monkeypox virus (MPXV) has spread globally, posing a severe challenge to global public health. This study systematically evaluated the aerosol shedding dynamics of the epidemic Clade IIb MPXV strain in infected young rabbits, along with its direct contact and airborne transmission potential among them. We found that young rabbits could be experimentally infected with MPXV, exhibiting distinct pathogenic features and viral shedding patterns. Young rabbits infected with MPXV shed the virus through nasal secretions and exhaled aerosols, peaking at 7 dpi. In total, 89–95.8% of virus-laden respiratory particles had a diameter ≥4.7 μm. Notably, MPXV can be efficiently shed and transferred among young rabbits through direct contact and airborne routes. The nasal secretions and exhaled virus particles from donor rabbits can be contacted or inhaled by recipient rabbits. Large amounts of viral DNA were detected in the nasal wash of rabbits exposed to contact or airborne exposure. Furthermore, virus particles invade the lungs, causing pathological changes and disseminating them to multiple organs. However, no infectious virus was successfully recovered from these recipient rabbits, as their exposed or inhaled MPXV dose might have been below the MPXV’s minimum infectious dose for young rabbits. These findings indicate that although the airborne transmissibility of the current MPXV strain is relatively limited, inhalation of viral particles following airborne exposure can still result in bodily damage. Continuous monitoring of MPXV transmissibility and mutation evolution is imperative to prevent efficient respiratory aerosol transmission, which guides global monkeypox prevention and control strategies.

## 1. Introduction

The monkeypox virus (MPXV) is classified within the genus *Orthopoxvirus*, family Species *Orthopoxvirus monkeypox*, and was initially identified in non-human primates [1]. Similar to variola virus and vaccinia virus, monkeypox virus (MPXV) is an enveloped, double-stranded DNA virus. As a zoonotic pathogen, it can infect both humans and animals, causing mpox disease [2]. Patients infected with MPXV typically present with prodromal symptoms initially, including fever, lymphadenopathy, myalgia, fatigue, headache, and back pain. Several days after the onset of initial symptoms, patients typically develop skin rashes or mucosal lesions. Skin or mucosal lesions may occur on the extremities, genitals, and face, and can spread to the entire body in severe cases, with the number of lesions ranging from several to several thousand [3].

Monkeypox has spread on a global scale and currently affects 110 countries and regions [4]. As of March 2025, the World Health Organization has received over 138,000 reports of confirmed human cases, with a cumulative death toll of 317 [5]. According to Centers for Disease Control and Prevention of the United States (CDC), MPXV can be transmitted between humans through both sexual and non-sexual contact routes [6]. In terms of non-sexual transmission, the primary routes include direct contact with the rash, ulcers, or scabs of an infected individual; indirect contact with contaminated items or surfaces; and transmission via respiratory droplets or secretions from infected individuals. Sharing items with an infected person or cohabiting in the same living environment also increases the risk of MPXV transmission through contact. All of these transmission routes involve direct physical contact with infected animals or humans [7,8]. However, the current evaluation of MPXV’s transmissibility is primarily based on case investigations or epidemiological survey results during outbreaks, and its transmissibility still lacks support from more direct experimental data.

The continuous mutation, evolution and spread of MPXV have currently classified it into two major evolutionary clades, Clade I and Clade II, which are further subdivided into subclades Ia, Ib, IIa and IIb [9,10]. Since May 2022, the Clade IIb MPXV strain has emerged as the predominant strain driving the global monkeypox outbreak [11,12]. However, comprehensive data regarding the transmission characteristics of Clade IIb MPXV remain unreported. Previous studies have shown that young rabbits are ideal animal infection models for MPXV, but the transmissibility of this virus among individual young rabbits remains unclear [13]. In this study, we established a viral infection model in young rabbits via intranasal inoculation with Clade IIb MPXV, and evaluated the transmissibility of MPXV among young rabbits, including direct contact transmission and airborne transmission. In addition, this study analyzed the pattern of airborne virus shedding in young rabbits after MPXV infection. This study fills the research gap regarding the transmission characteristics and airborne virus shedding patterns of Clade IIb MPXV, providing important scientific evidence for a deeper understanding of the transmission risk of MPXV among susceptible animals and natural host populations.

## 2. Materials and Methods

### 2.1. Ethics Statement

All animal experiments were conducted strictly in accordance with the animal welfare guidelines (Terrestrial Code in 2004) of the World Organisation for Animal Health (WOAH). The use of experimental animals has been approved by the Animal Ethics Committee of Hebei Agricultural University (Document number of approval: 2024097l) and the Institutional Animal Care and Use Committee of Changchun Veterinary Research Institute, Chinese Academy of Agricultural Sciences (approval number: IACUC-ms-11-2023-040). In addition, all procedures involving MPXV were conducted in an Animal Biosafety Level 3 (ABSL-3) laboratory at the Changchun Veterinary Research Institute, Chinese Academy of Agricultural Sciences.

### 2.2. Virus and Cell

The hMpxV/China/GZ8H-01/2023 (Genebank: PP778666.1) strain of the monkeypox virus was generously provided by the Eighth Affiliated Hospital of Guangzhou Medical University and subsequently propagated in Vero E6 cells. This strain was isolated from a patient in Guangzhou, China. The master seed virus, obtained through proliferation, is stored at −80 °C. Vero-E6 cells were cultured in a humidified incubator set at 37 °C with 5% CO_2_. The culture medium was composed of Dulbecco’s Modified Eagle Medium (DMEM, Sigma-Aldrich, Burlington, MA, USA) supplemented with 10% fetal bovine serum (FBS, Invitrogen, Carlsbad, CA, USA), 50 U/mL penicillin, and 50 μg/mL streptomycin.

### 2.3. Animals and Viral Challenge

The experimental animals utilized in this study were 20-day-old male New Zealand white rabbits with a body weight ranging from 380 to 420 g, purchased from Sipeifu (Beijing, China). The young rabbits were accompanied by their mothers throughout the entire duration of the experiment. No observations were made on the mother rabbits in this study. Animals were maintained under strictly controlled environmental conditions, with the temperature set at 24 ± 2 °C, relative humidity ranging from 40% to 70%, and a 12 h light/dark cycle. Prior to the initiation of the experiment, these animals were randomly assigned to each experimental group and acclimated in an ABSL-3 facility for 2–3 days. The virus inoculation experiment was conducted under isoflurane inhalation anesthesia. Following confirmation of successful anesthesia, the animals were intranasally inoculated with 10^5.85^ PFU MPXV in DMEM.

### 2.4. Pathogenicity of Monkeypox Virus in Young Rabbit

To investigate the pathogenicity of MPXV in young rabbits, we monitored the body weight changes and survival rates of MPXV-infected young rabbits. Sixteen young rabbits were randomly divided into an infected group and a control group, with 8 rabbits in each group. The infected group was intranasally inoculated with 10^5.85^ PFU of MPXV, while the control group received intranasal inoculation with an equal volume of DMEM. Body weight and survival rates of the young rabbits were recorded daily within 13 days post-inoculation. To elucidate the tissue distribution characteristics of MPXV in young rabbits and the associated pathological damage resulting from infection, three young rabbits were assigned to the infected group, and another three young rabbits served as the control group. At 7 dpi, the animals were deeply anesthetized and euthanized by exsanguination. Tissue samples including nasal turbinate, trachea, heart, lung, liver, kidney, spleen, intestine, blood, and testis (together with epididymis) were collected from animals in the infected group. These samples were promptly immersed in 1 mL of PBS containing 2% penicillin-streptomycin for subsequent processing. Subsequently, the tissues were homogenized and centrifuged, and the resulting supernatant was collected for further analysis. The supernatants of blood or tissue homogenates were subjected to 10-fold serial dilution. A standard orthopoxvirus plaque assay was performed on Vero-E6 cell monolayers. The cells were then incubated at 36 °C in a humidified atmosphere with 6% carbon dioxide (CO_2_) for 72 h. At the end of incubation, the cells were stained with a 2× crystal violet solution supplemented with formalin to visualize the plaques, thereby determining the presence and quantity of viable viruses.

In addition, at 7 dpi, tissue samples were collected from 3 young rabbits in both the infected group and the control group. These samples were fixed with paraformaldehyde, followed by paraffin embedding and sectioning. Hematoxylin-eosin (HE) staining was then performed for histopathological examination to further evaluate the pathological damage to young rabbit tissues caused by MPXV infection.

### 2.5. Transmissibility of Monkeypox Virus in Young Rabbit

To assess the transmissibility of MPXV through direct contact in young rabbits, this study inoculated 3 young rabbits with MPXV via nasal drop infection, which served as donor animals. After 24 h, 3 donor rabbits were transferred to a clean cage and co-housed with 3 naïve young rabbits (recipient rabbits) and mother rabbit. Donor rabbits and recipient rabbits can be in direct contact to study the direct contact transmissibility of MPXV among rabbits. For the assessment of airborne transmissibility, 24 h after infection, 3 donor young rabbits and mother rabbit were transferred to a wire mesh cage and placed adjacent to another wire mesh cage containing 3 recipient rabbits and another mother rabbit. The donor rabbit and recipient rabbit cages were kept at a distance of 2.4 inches apart, which prevented direct contact but allowed for interaction through respiratory transmission to study the airborne transmissibility of MPXV among rabbits.

Nasal washes were collected from both donor and recipient rabbits on days 1, 3, 5, 7, 9, 11, and 13 post-infection (dpi), which correspond to days 0, 2, 4, 6, 8, 10, and 12 post-exposure (dpe). At 12 dpe, the animals were deeply anesthetized and euthanized by exsanguination. The internal organs of recipient rabbits were collected, including the nasal turbinate, trachea, lungs, heart, liver, spleen, kidneys, intestine, and testis (together with epididymis). The PFU of nasal washes was determined, and the viral load were quantitatively analyzed using standard methods. Organs were homogenized in 1 mL of PBS supplemented with 2% penicillin-streptomycin. Following collection of the supernatant, the PFU was determined, and viral load was quantified.

### 2.6. Collection of Exhaled Viral Aerosols from Rabbit

The 3 young rabbits were intranasally inoculated with MPXV. Exhaled breath samples from the young rabbits were collected on 1, 3, 5, 7, 9, 11, and 13 dpi using the Anderson six-stage impactors (TE-20-800; Tisch Inc., Cleves, OH, USA). The collection parameters were set at a flow rate of 28.3 L per minute for a duration of 60 min per session. Anderson six-stage impactors can classify aerosol particles in the collected oral and nasal exhaled breath into six size ranges (>7 μm, 4.7–7 μm, 3.3–4.7 μm, 2.1–3.3 μm, 1.1–2.1 μm, and 0.65–1.1 μm) based on their aerodynamic diameters. Aerosol particles within each size range are subsequently collected onto separate, pre-sterilized gelatin filters (Sartorius, Göttingen, Germany), as previously described [14]. The aerosol samples were used for the quantitative detection of viral nucleic acid.

### 2.7. Viral Nucleic Acid Detection

DNA was extracted using the TIANamp Virus DNA/RNA Kit (TIANGEN, Beijing, China) and quantified by quantitative real-time PCR (qPCR) using the One Step PrimeScript^TM^ RT-PCR Kit (TaKaRa, Kyoto, Japan), following the manufacturers’protocols. The primers and probe targeting the *F3L* gene of monkeypox virus were as follows: [Forward: 5′-CTCATTGATTTTTCGCGGGATA-3′; Reverse: 5′-GACGATACTCCTCCTCGTTGGT-3′; Probe: 5′ FAM-CATCAGAATCTGTAGGCCGT-3′ TAMRA]. In qPCR analysis, the standard plasmid harboring the *F3L* gene was used to establish a standard curve, enabling the absolute quantification of viral copy numbers. The qPCR was run on the ABI 7500 System (ThermoFisher, Waltham, MA, USA).

### 2.8. Statistical Analysis

Data were analyzed using GraphPad Prism 10 software (San Diego, CA, USA). One-way analysis of variance (ANOVA) was used to determine statistically significant differences between groups. *p*  <  0.05 was considered statistically significant. All assays were performed in triplicate and are representative of at least three independent experiments.

## 3. Results

### 3.1. Body Weight Changes and Survival Rate of Rabbit Following MPXV Infection

**Figure 1:**
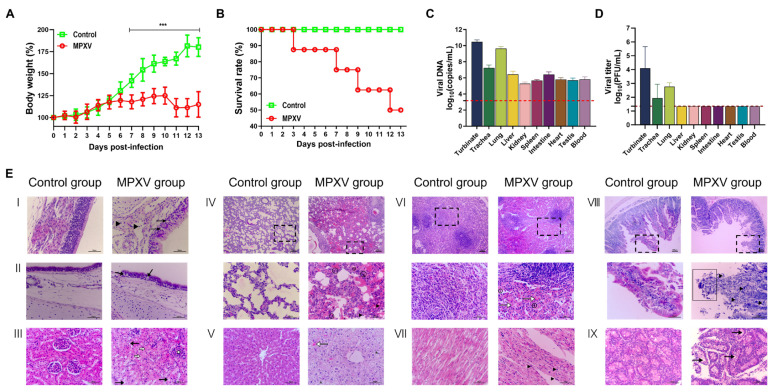
Pathogenicity of clade IIb MPXV in young rabbits. The 20-day-old young rabbits were intranasally inoculated with DMEM containing or not containing 10^5.85^ PFU MPXV, and their body weight and survival rate were monitored continuously for 13 days. At 7 dpi, tissue samples from the nasal turbinate, trachea, lungs, liver, kidneys, spleen, small intestine, heart, testes, and epididymis, as well as blood samples, were collected from young rabbits to assess viral load and detect the presence of infectious virus. (**A**) Body weight changes in rabbits (n = 8). “***”: *p* < 0.0001. (**B**) Survival rate (n = 8). (**C**) Viral load and (**D**) virus titer in various organs of rabbits following MPXV infection. Results are expressed as mean ± SD; n = 3. The red dashed line represents the minimum detection limit. Tissue samples were fixed in formalin, embedded in paraffin, and subjected to pathological examination using hematoxylin-eosin (H&E) staining for (**E**) histopathological analysis. The numbers in the figure represent the following, respectively: I: Nasal mucosa; II: Trachea; III: Kidney; IV: Lung; V: Liver; VI: Spleen; VII: Heart; VIII: Small intestine; IX: Epididymis. Dashed boxes indicate locally magnified areas. (▲): Lymphocyte infiltration; (→): Vacuolar structures in intercellular spaces or cellular vacuolar degeneration; (〇): Hemosiderin; (⇾): Congestion; (*): Cellular ballooning degeneration (Located in **E**,V); (➪): Cellular necrosis; (✩): Mild congestion and swelling of glomeruli; (□): Epithelial exfoliation at the tips of intestinal villi. Scale bars represent 50 μm or 100 μm.

We assessed the pathogenicity of clade IIb MPXV in young rabbits and found that MPXV infection not only suppressed weight gain but also induced lethal outcomes. The body weight of the control group increased steadily throughout the observation period, reaching 180% of the initial weight by the end of the experimental duration. The body weight gain in the MPXV-infected group was significantly reduced compared to the Control group (*p* < 0.0001). Weight gain in the MPXV-infected group plateaued at 6 dpi and began to decrease at 10 dpi, suggesting that MPXV infection negatively impacts the growth of young rabbits (Figure 1A). The survival rate of rabbits in the control group remained consistently at 100%. In contrast, the survival rate of the MPXV-infected group began to decline from 3 dpi, reaching a mortality rate of up to 50% by 12 dpi. This trend indicates that MPXV exhibits high pathogenicity in young rabbits (Figure 1B).

We evaluated the tissue distribution of MPXV in young rabbits and found that MPXV rapidly disseminated to the respiratory organs following intranasal inoculation in young rabbits. The highest viral loads were observed in the nasal turbinate and lung tissues, reaching 2.99 × 10^10^ copies/mL and 4.45 × 10^10^ copies/mL (10^4.10^ PFU/mL and 10^2.75^ PFU/mL), respectively. High viral loads were also detected in the trachea, with a viral load of 1.76 × 10^7^ copies/mL (10^1.95^ PFU/mL). In addition to the respiratory organs exhibiting highly active viral replication, MPXV DNA was also detected in organs including the liver, kidney, spleen, intestine, heart, and testicle. Among these, the viral loads in the liver and intestine were relatively higher, being 2.79 × 10^6^ copies/mL and 2.68 × 10^6^ copies/mL, respectively. However, infectious virus was not successfully detected in these tissues. Furthermore, we observed that high viral loads, reaching up to 6.67 × 10^7^ copies/mL, were also present in the blood of young rabbits. Therefore, we propose that following intranasal inoculation of MPXV in young rabbits, the virus may not only invade the respiratory tract and replicate extensively, but also disseminate to multiple organs via the circulatory system.

We further examined the histopathological alterations in the organs of young rabbits infected with MPXV (Figure 1E). No significant pathological damage was detected in the organs of young rabbits in the control group. In contrast, following infection with MPXV, young rabbits exhibited varying degrees of pathological damage across multiple tissues and organs. (Figure 1(EI)): The nasal mucosal epithelium in the control group was intact with a normal structure. In contrast, the infected group showed exfoliation of the mucosal epithelium, a significant reduction in the number of epithelial cells, narrowing of the entire mucosa, occasional vacuolar structures (→), and infiltration of lymphocytes (▲) in the submucosa. (Figure 1(EII)): The tracheal mucosal epithelium in the control group appeared relatively normal. In the infected group, the tracheal mucosa occasionally exhibited discontinuity, with sparsely arranged epithelial cells and vacuolar structures present in the intercellular spaces (→). (Figure 1(EIII)): The kidney structure in the control group was normal, with no histopathological changes observed in the renal corpuscles or renal tubules in the cortical region. In the infected group, the renal glomeruli (✩) showed slight congestion and swelling, the lumen of the Bowman’s capsule was narrowed, the structure of the renal tubules was indistinct, and the renal tubular epithelial cells exhibited swelling, necrosis (➪), and vacuolar degeneration (→). (Figure 1(EIV)): The lung tissue structure in the control group was relatively normal. In the infected group, the pulmonary interstitium was significantly widened, the alveolar walls were thickened to varying degrees, the interstitial capillaries were dilated and congested, some alveolar lumens contained serous exudates with inflammatory cell infiltration (▲), and hemosiderin in the alveolar septa was significantly increased. (Figure 1(EV)): In the control group, the structure of hepatocytes, hepatic cords, and hepatic sinusoids in the liver was relatively normal. In the infected group, the hepatic cords were arranged in a disorganized manner, occasional congestion was observed in the hepatic sinusoids (⇾), and hepatocytes showed obvious swelling with pale-stained cytoplasm, presenting ballooning degeneration (*). (Figure 1(EVI)): The red pulp and white pulp of the spleen in the control group showed relatively normal structures. In the infected group, there was no significant change in the size of the white pulp; however, the red pulp exhibited congestion, increased hemosiderin (〇), and widened and congested sinusoids (⇾). (Figure 1(EVII)): In the control group, the myocardium was uniformly stained overall, with myocardial fibers arranged neatly and orderly, and there were certain relatively wide gaps between muscle cells and muscle fibers. In the infected group, the myocardial fibers of the heart were arranged in a disorderly manner, with vacuolization observed in the cytoplasm of myocardial cells, and occasional infiltration of inflammatory cells (▲). (Figure 1(EVIII)): The intestinal villi structure in the control group was relatively intact. In the infected group, the number and height of intestinal villi were significantly reduced, with severe exfoliation of the epithelium at the villus tips (□), lymphocyte infiltration in the lamina propria (▲), a marked increase in intestinal glands, and thinning of the muscular layer. (Figure 1(EIX)): In the control group, the epithelium of the epididymal ducts was intact, with principal cells arranged closely in a tall columnar manner. In the infected group, numerous vacuoles (→) were observed between the epididymal duct epithelial cells and within the ductal lumen, and the interspace between the epididymal ducts was widened.

### 3.2. Direct Contact and Airborne Transmissibility of MPXV in Rabbit

**Figure 2:**
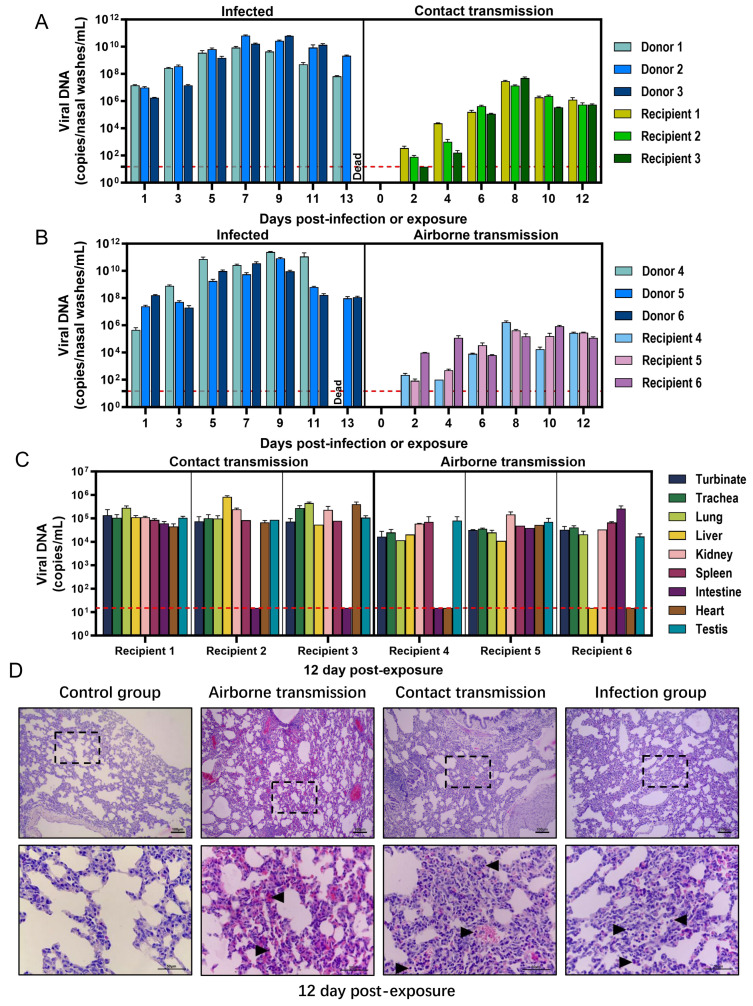
The transmissibility of Clade IIb MPXV among young rabbits. To evaluate direct contact transmissibility, 3 donor rabbits and 3 recipient rabbits were co-housed in the same cage, enabling direct contact between the two groups of animals. For assessing airborne transmissibility, 3 donor rabbits and 3 recipient rabbits were housed separately in two independent cages, with a distance of 2.4 inches between the two cages. No direct contact was possible between the animals; however, respiratory interaction could occur through the ventilation holes. From 1 to 13 dpi (0–12 dpe), nasal wash samples were collected every other day, and the viral load was determined. (**A**) Viral loads in nasal washes from the infected group and the contact transmission group; (**B**) Viral loads in nasal washes from the infected group and the airborne transmission group. Following the completion of nasal wash collection at 13 dpi (12 dpe), multiple tissues and organs were collected from the recipients in both the contact transmission group and the airborne transmission group, and (**C**) the viral load was subsequently quantified. The red dashed line represents the minimum detection limit. In addition, HE staining was performed on lung tissues to evaluate (**D**) the pathological changes in the recipient rabbits of the contact transmission group and the airborne transmission group. (▲): Lymphocyte infiltration. Scale bars represent 50 μm or 100 μm. The position of the dotted-line box represents the field of view that is further magnified.

To evaluate the contact transmissibility of MPXV among rabbit populations, 3 young rabbits were selected and intranasally inoculated with 10^5.85^ PFU of the virus, designated as donor rabbits. At 1 dpi (0 dpe), the 3 donor rabbits were transferred to the cage housing another 3 recipient rabbits, allowing for direct contact under experimental conditions. We found that the viral load in the nasal washes of donor rabbits showed a trend of first increasing and then decreasing, and remaining at a high concentration throughout the period (Figure 2A). The highest viral load appeared at 7 dpi, reaching 6.60 × 10^10^ copies/mL. This indicates that all donor rabbits were infected and continuously shed the virus into the external environment through the nasal route. Viral DNA was successfully detected in nasal wash samples obtained from recipient rabbits that had direct contact with donor rabbits (Figure 2A), and the viral load demonstrated a trend of initial increase followed by a decline. During 0–4 dpe, the viral load remained at a relatively low level; it significantly increased from 6 dpe and reached its peak at 8 dpe, averaging 3.05 ± 1.78 × 10^7^ copies/mL. The above results indicate that MPXV can shed from infected young rabbits and transfer to naïve animals through direct contact.

To evaluate the aerosol transmissibility of MPXV among young rabbit populations, the experiment was conducted by housing 3 donor rabbits and 3 recipient rabbits in two adjacent wire cages at 1 dpi (0 dpe). The two cages were separated by 2.4 inches, ensuring that no direct contact could occur between donor and recipient rabbits while allowing respiratory interaction through the ventilation holes of the cages. We found that the viral shedding of donor rabbits in the airborne transmission group was similar to that of donor rabbits in the direct contact transmission group (Figure 2B). Viral DNA was also detectable in nasal wash samples from recipient rabbits exposed via air. The viral load exhibited a trend of first increasing and then decreasing, with a peak reaching 8 dpe (7.56 ± 8.20 × 10^5^ copies/mL). These results indicate that MPXV can also shed from infected young rabbits and transfer to naïve animals through exhaled aerosols.

At 12 dpe, all recipient rabbits survived, with their internal organs collected for viral load detection and pathological changes in the lung tissues observed. As shown in Figure 2C, in the recipient rabbits exposed through direct contact, viral DNA was detected in various tissues including the nasal turbinate, trachea, lungs, liver, kidneys, spleen, heart and reproductive organs, with viral loads ranging from 4.5 × 10^4^ to 8.31 × 10^5^ copies/mL. Additionally, viral DNA was occasionally detected in the small intestine tissues of some samples. In the air-exposed recipient rabbits, viral DNA could be stably detected in most organs such as the nasal turbinate, trachea and lungs, with viral loads ranging from 1.09 × 10^4^ copies/mL to 2.59 × 10^5^ copies/mL. The results further demonstrate that MPXV can shed and transfer among young rabbits through direct contact or airborne routes. Upon entry into the respiratory tract of the recipient rabbits, the virus can subsequently disseminate to internal organs through the circulatory system.

As illustrated in Figure 2D, compared with the control group, the lung tissues of recipient rabbits exposed to air exhibited marked widening of the pulmonary interstitium. Concurrently, significant hyperplasia of alveolar epithelial cells, thickening of alveolar walls, capillary congestion, and mild inflammatory cell infiltration (▲) were observed. The lung tissues of the receptor rabbits exposed to contact exhibited more severe pathological damage, characterized by further widening of the pulmonary interstitium, accompanied by marked capillary dilation and congestion, as well as a significant increase in inflammatory cell infiltration (▲).

### 3.3. Concentrations and Particle Size Distribution of Viral Aerosols Exhaled by MPXV-Infected Rabbit

**Figure 3:**
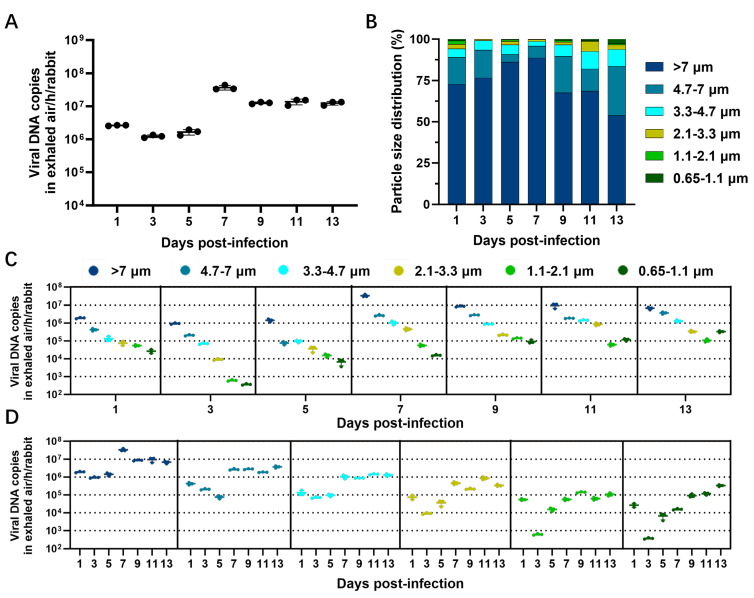
Viral concentration and particle size distribution of virus-laden particles in the exhaled aerosol from MPXV-infected rabbits. At 1–13 dpi, viral aerosols exhaled by MPXV-infected rabbits were collected. The sampler can collect virus-laden particulates in the virus aerosol into six presterilized gelatin filters according to the aerodynamic diameters of the particulates (>7 μm, 4.7–7 μm, 3.3–4.7 μm, 2.1–3.3 μm, 1.1–2.1 μm, 0.65–1.1 μm). (**A**) The total viral concentration in the aerosols exhaled by MPXV-infected rabbits. (**B**) The size distribution of virus-laden particles in viral aerosols. (**C**) Viral concentration in virus-laden particles classified by infection time. (**D**) Virus concentration in virus-loaded particles classified by particle size. The data in panels (**A**,**C**,**D**) were presented as mean ± standard deviation (SD). The detection limit is 6 copies in exhaled air/hour/rabbit.

On the assessment of the airborne transmissibility of MPXV, viral DNA was detected in the nasal washes and multiple organs of all recipient rabbits, indicating that MPXV-infected young rabbits can release the virus into the ambient air through aerosols. Thus, we further analyzed the kinetic characteristics of virus release via the aerosol route in MPXV-infected rabbits. We found that the total concentration of viral particles in the exhaled viral aerosols gradually increased at the early stage of infection and then slightly decreased (Figure 3A). From 1 to 5 dpi, the viral shedding was relatively low, ranging from 1.35 × 10^6^ copies/h/rabbit to 2.66 × 10^6^ copies/h/rabbit. At 7 dpi, the total concentration peaked, reaching 4.41 × 10^7^ copies/h/rabbit, which was significantly higher than at all other time points. Subsequently, the total concentration exhibited a slight decline but remained at a consistently high level, ranging from 1.33 × 10^7^ to 1.54 × 10^7^ copies/h/rabbit. However, no infectious virus was detected in any of the exhaled viral aerosol samples.

We further analyzed the particle size distribution of virus-laden particles in the viral aerosol. We found that the majority of the virus was present in coarse particles with a diameter >4.7 μm (Figure 3B). The proportion of coarse particles with a diameter >4.7 μm was consistently the highest, ranging from 89% to 95.8% from 1 to 9 dpi. In contrast, the proportion of particles with a diameter <4.7 μm is relatively low, and the smaller the particle size, the lower the proportion.

Figure 3C,D presents detailed data on the virus concentration in particles of different size ranges. Analysis revealed that larger particle sizes of virus-laden particles were associated with higher viral concentrations (Figure 3C). Throughout the entire observation period, the viral concentration in particles with a diameter >7 μm remained at a relatively high level, ranging from 1.03 × 10^6^ copies/h/rabbit to 3.92 × 10^7^ copies/h/rabbit (Figure 3D). For particles with a diameter of 4.7–7 μm, the viral concentration was relatively low from 1 to 5 dpi, but significantly increased after 7 dpi, reaching 1.9 × 10^6^ to 3.7 × 10^6^ copies/h/rabbit. For particles in the size range of 3.3–4.7 μm, the viral concentration can reach 8.73 × 10^5^ copies/h/rabbit to 1.4 × 10^6^ copies/h/rabbit after 7 dpi. In contrast, for particles in the size range of 0.65–3.3 μm, the viral concentration also gradually increased with the infection time; however, the overall level remained low and never exceeded 10^6^ copies/h/rabbit.

## 4. Discussion

This study found that 20-day-old young rabbits could be experimentally infected with Clade IIb MPXV. After nasal drop infection, the weight gain of the young rabbits stopped and the mortality rate was as high as 50%. MPXV can disseminate throughout the body of young rabbits via the bloodstream, resulting in multi-organ damage. Viral nucleic acids can be detected in various tissues and organs such as the heart, liver, spleen, lungs, kidneys, brain, intestines, reproductive organs, trachea and nasal conchae, but infectious viruses are only found in respiratory tract tissues. Previous studies established an MPXV infection model in young rabbits using 10-day-old subjects [13]. However, the limited mobility of 10-day-old rabbits, along with the high mortality risk associated with nasal wash sample collection, complicates the assessment of viral transmissibility. To address these limitations, this study utilized 20-day-old young rabbits. Furthermore, experimental results indicated that 20-day-old rabbits retained the essential infection characteristics of MPXV.

Studies from the last century have demonstrated that among littermate rabbits co-housed with adult female rabbits, those inoculated with the Copenhagen reference strain of MPXV via the oral or intranasal route are capable of transmitting the virus to uninfected littermates and the adult female rabbit through contact [15]. Infectious virus (10^2^ PFU/mL) could be detected in the internal organs of young rabbits infected through exposure. Another study suggested that the transmission among the littermates is caused by airborne droplet [16]. The transmission route and efficiency of MPXV in the rabbit population remain unknown. This study excluded the possible virus transmission (sucking the same maternal teat) that might occur during the suckling process of young rabbits. Young rabbits infected with Clade IIb MPXV by nasal drops were co-housed or housed separately with naïve young rabbits, allowing donor rabbits and recipient rabbits to have direct contact or only air contact, in order to evaluate the direct contact and airborne transmissibility of MPXV among young rabbits. We observed that MPXV could be efficiently shed and transferred through contact and air among young rabbits, and caused lung injury in recipient rabbits. However, we successfully detected infectious virus only in the donor rabbit, and not in the recipient rabbit (Supplementary Figure S1). This indicates that the transferred virus did not cause a substantial infection. We speculate that this may be attributable to a high minimum infectious dose of Clade IIb MPXV in the young rabbit model. Our previous studies have demonstrated that the minimum infectious dose (MID) of the hMpxV/China/GZ8H-01/2023 strain (the same as the one used in this study) required to initiate mild infections in young rabbits is 10^4.5^ PFU [13]. During 3–11 dpi in donor rabbits, the titer of infectious virus shed remained below this MID at all time points except for the peak viral shedding phase (7 dpi) (Supplementary Figure S1).

**Figure 4:**
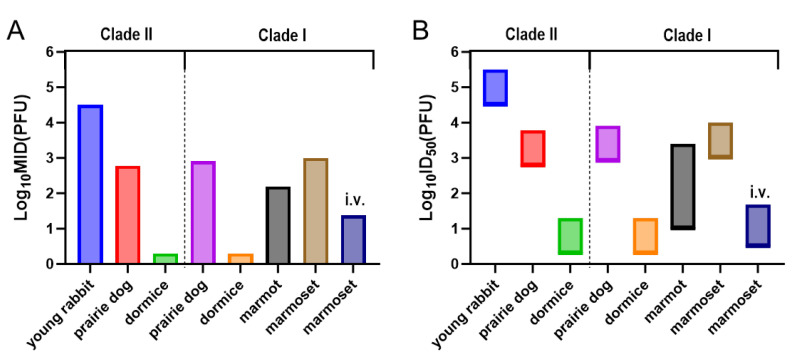
The minimum infectious dose (MID) and the range of ID_50_ for susceptible animals infected with MPXV. (**A**) MID; (**B**) ID_50_. The infection rates of susceptible animals infected with different doses of MPXV were obtained through literature retrieval. The lowest dose that can cause an infection rate greater than 0% was taken as the MID and the lower limit of ID_50_, and the lowest dose that can cause an infection rate of 100% was taken as the upper limit of ID_50_. i.v.: Intravenous inoculation.

The susceptibility of animals to pathogenic microorganisms, to a certain extent, determines the infection and transmission efficiency of these pathogens. However, the susceptibility of young rabbits to MPXV appears to be relatively low, making it difficult to induce infection in recipient rabbits. Studies have demonstrated that intranasal inoculation of young rabbits with 10^3.5^ PFU of Clade IIb MPXV does not induce significant clinical signs, and no detectable viral DNA is present in the body [13]. Upon inoculated with 10^4.5^ PFU MPXV, the young rabbits only showed mild tissue damage and slight weight loss. Only 33.3% (1 out of 3) of the young rabbits had viral DNA detected in their organs (spleen). All young rabbits (3 out of 3) showed obvious infection symptoms when infected with a dose of 10^5.5^ or 10^6.5^ PFU MPXV. This indicates that young rabbits have a relatively high infection threshold, where infection may only be induced when the infectious dose reaches 10^4.5^ PFU, while the infection rate increases significantly when the dose reaches 10^5.5^ PFU (Figure 4). MPXV can be transmitted among its natural hosts, such as the black-tailed prairie dog and the rope squirrel, and the presence of infectious virus has been confirmed in the recipient animals [17,18,19]. A dose of 10^2.78^ PFU of Clade II MPXV can infect 25% (1 out of 4) of black-tailed prairie dogs [20]. A dose of 10^3.78^ PFU of Clade II MPXV can infect 75% or 100% (3 out of 4 or 4 out of 4) black-tailed prairie dogs [19,20]. A slightly higher dose, 10^3.90^ PFU of Clade II MPXV, is capable of infecting 100% (14 out of 14) of black-tailed prairie dogs [21]. These findings indicate that prairie dogs exhibit significantly higher susceptibility to Clade II MPXV infection compared to young rabbits. Although no direct data on the infection threshold of rope squirrels has been reported, as a natural host of MPXV, infected rope squirrels can transmit the MPXV to primates (including humans) through predation or contact [22,23], and their susceptibility appears to be greater compared to that of young rabbits, which are considered non-natural hosts. Other natural hosts of MPXV, such as dormice and marmots, show a significantly higher susceptibility than young rabbits, although there is no evidence yet that MPXV can be transmitted among their populations. A dose of 10^0.3^ PFU of Clade I or II MPXV can induce a 40% mortality rate in dormice, and a dose of 10^2.3^ PFU can results in a 100% mortality rate [24]. A dose of 10^2.2^ PFU of Clade I MPXV can cause infection-related symptoms in 50% of marmots (2 out of 4) [25,26]. Natural transmission of MPXV can also occur among non-human primates [27], and these animals exhibit a higher susceptibility to MPXV than young rabbits [28,29]. Detailed data are available in Table 1.

To some extent, the particle size of virus-laden particulate matter influences the efficiency of airborne transmission of pathogenic microorganisms. Traditionally, it is believed that larger particles (>5 μm), such as droplets, settle more quickly, and the pathogenic microorganisms contained therein have a limited transmission distance. Aerosols with smaller particle sizes (<5 μm) can remain suspended in the air for extended periods and transmit over long distances, potentially leading to long-range infections [30]. Particles <5 μm are smaller in size and thus more likely to penetrate deep into the lungs of animals, thereby inducing infections; while particles >5 μm are more likely to deposit in the upper respiratory tract such as the nasal cavity and trachea under gravitational influence, thus failing to reach the deep lung regions [31,32]. In this study, we found that MPXV-infected young rabbits can exhale a substantial quantity of virus-laden particles into the environment. These particles are predominantly coarse particles >4.7 μm. Consequently, the risk of airborne transmission of MPXV is primarily attributed to droplet transmission. We found that the viral load in the nasal washes of recipient rabbits exposed to airborne particles from donor rabbits maintained at a distance of 2.4 inches was lower than that of recipient rabbits exposed directly, and the lung damage was also less severe. This indicates that the transmission of virus-laden particles exhaled by young rabbits is significantly influenced by distance. When the donor rabbits and recipient rabbits are kept at an interval of 2.4 inches, the amount of virus entering the recipient rabbits’ respiratory tracts is significantly reduced.

In conclusion, although the transmission of the epidemic Clade IIb MPXV strain among young rabbits is ineffective, the virus can still shed from infected individuals and transfer within the population. The virus particles can enter the respiratory tract of recipient young rabbits through direct contact or airborne transmission, causing lung damage. As the virus penetrates deeper into the respiratory tract, the virus particles also disseminate to various organs within the body through the bloodstream. Young rabbits infected with MPXV can shed a large amount of virus-laden particles into the ambient environment through exhaled breath. The particles are mainly coarse particles >4.7 μm. Although the distance of viral shedding and transfer is limited, the virus (shed from infected young rabbits) can still enter the respiratory tract of recipient young rabbits. As companion or economic animals, rabbits often come into contact with humans. These virus-laden particles also pose a potential threat to public health and are a potential risk factor for the transmission of MPXV from animals to humans. It is recommended to formulate and implement an appropriate regulatory framework and enhance the monitoring of domestic and wild rabbit populations to effectively break the potential transmission chain from animals to humans. These findings provide important basis for strengthening the prevention and control strategies used for monkeypox.

## Figures and Tables

**Table 1:** Susceptibility and transmissibility of susceptible animals to MPXV.

Species	MID(log_10_ PFU)	ID_50_(log_10_ PFU)	Inoculation Route	Clade	Strain	Determination of Infection	Transmissibility
young rabbit [13]	4.50	4.5~5.5	i.n.	II	hMpxV/China/GZ8H-01/2023	Weight changes and DNA detection results	Ineffective
Prairie dogs [19,20,21]	2.78	2.78~3.78	i.n.	II	MPXV-USA-2003-044	Clinical manifestations and antibody seroconversion	100% contact transmission efficiency [18,19]
	2.91	2.91~3.91	i.n.	I	MPXV-ROC-2003-358	Clinical manifestations and antibody seroconversion	17% airborne transmission efficiency [19]
dormice [24]	0.30	0.3~1.3	i.n.	II	MPXV-COP-58	Mortality rate and weight	unknown
	0.30	0.3~1.3	i.n.	I	MPXV-ZAI-79	Mortality rate and weight	unknown
Marmot [25,26]	2.20	2.2 ± 1.2	i.n.	I	V79-1-005	Clinical manifestations	unknown
Marmoset [28,29]	3.00	3.0~4.0	i.n.	I	V79-1-005	Clinical manifestations	unknown
	<1.68	<1.68	i.v.	I	V79-1-005	Clinical manifestations	unknown
Rope squirrels	-	-	i.n.	I	MPXV-2003-Congo-358	-	Airborne and cross-species transmission cases exist. [17,22]
Baboon	-	-	i.m.	-	7-61 WRAIR	-	natural transmission cases exist [27]

## Data Availability

The original contributions presented in this study are included in the article/Supplementary Materials. Further inquiries can be directed to the corresponding authors.

## Supplementary Materials

The following supporting information can be downloaded at: https://www.mdpi.com/article/10.3390/microorganisms13092182/s1, Figure S1: Detection of infectious virus in donor rabbits and recipient rabbits.
