# Supplementary figures and images for: Transmissibility of Clade IIb Monkeypox Virus in Young Rabbits
Source: Microorganisms. 2025 Sep 18;13(9):2182. doi: 10.3390/microorganisms13092182 (PMC12472869; doi:10.3390/microorganisms13092182)

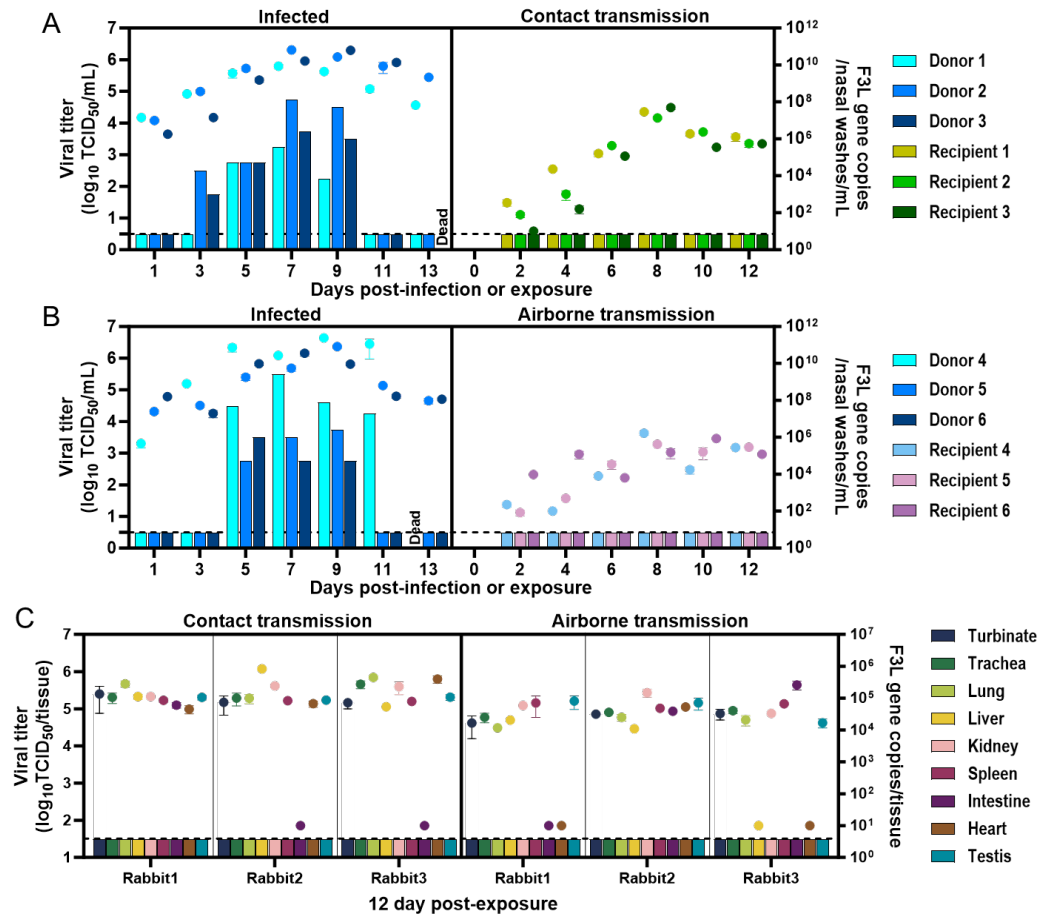

Supplementary Figure S1. Detection of infectious virus in donor rabbits and recipient rabbits.

Supplement: Supplementary file 1 [file microorganisms-13-02182-s001.zip › microorganisms-3821983-supplementary.pdf]
